# Distortion Prediction in Inconel-718 Part Fabricated through LPBF by Using Homogenized Support Properties from Experiments and Numerical Simulation

**DOI:** 10.3390/ma15175909

**Published:** 2022-08-26

**Authors:** Varun Ananda, Gurunathan Saravana Kumar, Rengaswamy Jayaganthan, Balamurugan Srinivasan

**Affiliations:** 1 Aero-Component and Systems Design Department, Honeywell Technology Solutions Lab Pvt. Ltd., Bangalore 560103, India; 2 Department of Engineering Design, Additive Manufacturing Group & Centre of Excellence in Materials and Manufacturing for Futuristic Mobility, Indian Institute of Technology Madras, Chennai 600036, India

**Keywords:** additive manufacturing, distortion, AM support structures, distortion simulation, homogenization of lattice supports

## Abstract

The Laser Powder-Bed Fusion (LPBF) process produces complex part geometry by selectively sintering powder metal layer upon layer. During the LPBF process, parts experience the challenge of residual stress, distortions, and print failures. Lattice-based structures are used to support overhang parts and reduce distortion; this lattice support has complex geometry and demands high computational effort to predict distortion using simulation. This study proposes a computational efforts reduction strategy by replacing complex lattice support geometry with homogenization using experimentally determined mechanical properties. Many homogenization models have been established to relate the lattice topology and material properties to the observed mechanical properties, like the Gibson–Ashby model. However, these predicted properties vary from as printed lattice geometry. In this work, the power-law relationship of mechanical properties for additively manufactured Inconel 718 part is obtained using tensile tests of various lattice support topologies and the model is used for homogenization in simulation. The model’s accuracy in predicting distortion in printed parts is demonstrated using simulation results for a cantilever model. Simulation studies show that computational speed is significantly increased (6–7 times) using the homogenization technique without compromising the accuracy of distortion prediction.

## 1. Introduction

LPBF is an Additive Manufacturing (AM) process technique that employs a highly focused laser as a moving heat source to scan the powder on the print bed and fuse it to produce the solid part. This process is repeated layer upon layer using multiple laser scans to achieve complex part geometry. During sintering, repeated heating and cooling develop a high thermal gradient on the printed part, causing residual stress build-up, leading to part distortion and print failure by support cracking or part delamination from the base plate [1,2]. The LPBF process uses an adequate amount of sacrificial support structures to overcome the challenges of part distortion and to support overhang features [3,4]. Supports also help to anchor the part to the base plate and provide a quick heat dissipation path for the sintering process [5]. Part distortion and overuse of support raise the cost of using powder bed AM technology in industrial applications. Thus, there is a need for a quick and accurate way of predicting distortion using FE simulation to evaluate geometric nonconformity before printing the part.

**Figure 1:**
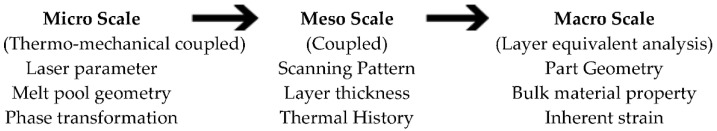
Multi-scale simulation approach in AM distortion analysis.

Several FE models have been developed for distortion prediction. The detailed micro-scale model developed by Fu et al. [6] and the thermo-mechanical model developed by Prabhakar et al. [7] use the concept of micro weld repeated along the entire scanning path with a layer by layer model. This approach provides accurate results. However, this may not be feasible for practical application with many layers of stacking and scanning because this model’s computational effort consumes days [8]. Many models are developed to increase computational speed, with the idea of thermally activating an entire layer at a time [9]. This approach neglects the effect of laser process parameters such as hatching space, scanning strategy, and layer rotation angles. Liang et al. [10] introduced a modified inherent strain homogenization method for FEA of AM part distortion, with significant reduction in simulation time. Another method is using a multi-scale approach [11,12], in which simulation is performed at three levels to achieve good distortion prediction accuracy and computational speed. Chen et al. [12] and Cheng et al. [13] demonstrated such approaches and compared experimental results with commercial FE simulation software (Simufact Additive^®^, Version 4.1, MSC Software Company, Hamburg, Germany) data. Most commercially available distortion simulation software uses a multi-scale simulation approach [14]. Generally, three levels of simulations are performed to achieve high accuracy and reasonable computational speed [15], as shown in Figure 1.

The micro-scale involves detailed thermo-mechanical simulation and is independent of part geometry and size. Meso-scale mainly considers the printing parameters such as laser scanning patterns, layer thickness, thermal history, and macro-scale and is dependent on the geometry of printed parts and material properties. The micro and mesoscale simulation steps depend on the material and process parameters inputs. However, the computational load on macro-scale simulation will vary with part complexity. In this work, the computational effort of simulating complex geometry in the case of lattice support is replaced with a simple homogenized solid block, loaded with an effective property that can mimic similar behavior of lattice. This approach will bring down the FE simulation effort from Macroscale.

Zeng et al. [16] explored a unique approach of creating a thermal model for complex lattice support geometry by replacing it with simple solid block geometry. The solid block was loaded with effective thermal conductivity. The findings of effective thermal conductivity for different support volume fractions have shown that computational speed increased drastically without much effect on solution accuracy. This study provides an approach to use effective properties of the LPBF supports to enhance computational speed. Literature provides numerous studies about an in-depth approach to numerical modeling and their validation of LPBF processes. However, it is observed that there are no experimental studies reported for evaluating the effective properties of complex lattice support structures. Thus, this study focuses on investigating the process of substituting the complex lattice support geometry with a homogenized continuum solid block using its effective properties measured from experimental characterization and analytical calculations, using specially designed tensile samples. Multiple simulation studies were conducted to understand the influence of various lattice support design parameters on the error between actual geometry simulation and homogenized model simulation. The proposed approach will be helpful in predicting the residual stress of metallic parts in industrial applications because very few variations of lattice supports are used in industrial part production and one-time characterization of all variations in standardized lattice support will bring down the computation load of repeated FEA efforts prior to new production part printing.

The remaining part of this manuscript is divided into three sections. The technique of substituting homogenized continuum support instead of complex lattice geometry, benchmarking of software’s accuracy, and the method used for evaluating effective property are explained in the work. The experimental characterization of lattice support results for its effective properties, such as effective modulus of elasticity (E) and effective thermal conductivity, are discussed. The FE simulation made with the use of the effective property scaling factor is compared with actual geometry and experimental results.

## 2. Materials and Methods

### 2.1. Material

Inconel 718 is selected in the study due to its vast application in the aerospace industry and higher tendency to part distortion [3]. All test samples were fabricated in the single print bed using the same process parameters to eliminate influences of process variation in the study. The powder material supplied from the Vacuum Induction Melting Argon Gas Atomizing process fulfilled the standard recommended material datasheet of EOS Nickel alloy 718 [17], shown in Table 1.

Virgin Inconel 718 powder was loaded into the machine to eliminate possible property variation due to recycled powder [18]. Its apparent density was 4.41 g/cm^3^ (inspected as per ASTM B212) and its tapped density 5.64 g/cm^3^ (examined as per ASTM B527). Its particle size distribution d10 of 20 μm, d50 of 31 μm and d90 of 43 μm (tested as per ASTM B822). M280 machine is used for printing, with standard EOS recommended process parameters for Inconel 718 (Parameter Set IN718_Performace 1.0) as specified in Table 2.

Based on findings from Klingbeil et al. [19], preheating will reduce the thermal gradient experienced by part and bring down distortion in the printed samples. Though preheating is not favorable for the current study, it was maintained at 80 °C to mimic the actual industrial part process parameters and realise the similar distortion observed in production.

### 2.2. Methodology

**Figure 2:**
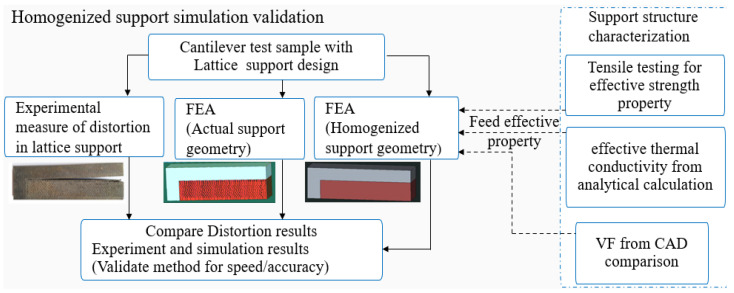
Flow chart for a homogenized support simulation approach.

The proposed methodology of substituting the homogenized property of lattice supports is achieved by finding the support structure’s effective strength and thermal property from experimental characterization and analytical calculations, as shown in Figure 2.

**Figure 3:**
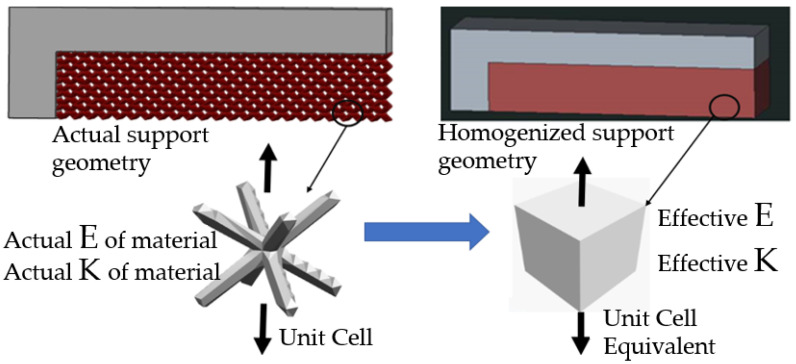
Replacing lattice support geometry with homogenized block support.

Sample representation of replacing actual lattice geometry with simple solid homogenized support is shown in Figure 3. The lattice support is assumed to be isometric, and the influence of directional behavior is not considered in this study. The strength of the support structure and heat transfer through the support structure significantly affects the distortion; this helps to reduce built-in stresses. A study by Zeng et al. [16] showed that the thermal conductivity of support structure is a combined effect of the volume fraction (VF) of the solid support and unsintered powder. In addition, they proposed an equation to find effective thermal conductivity as a function of support structure VF, the thermal conductivity of sintered metal, and unsintered powder:

(1) $$K_{\mathrm{effective}}=K_{\mathrm{solid}}\times \mathrm{VF}+{\;K}_{\mathrm{powder}}\times \left(1-\mathrm{VF}\right)=1,$$

The same approach is used in this study to find the effective thermal conductivity of support structures. Effective strength modulus $E_{\mathrm{Eff}}$ is found from experimental characterization. The Young’s modulus of full solid support was taken as 160 GPa, reported in the standard material datasheet of EOS Nickel Alloy IN718, made from EOS M280 machine using EOS recommended process parameters in the vertical Z direction [20]. Thermal absorptivity of 0.4, the thermal conductivity of solid sintered support taken as 9.1 W/mK and surrounding loose powder as 1.96 W/mK from a similar reference FE model were developed by Luo et al. [21] for the same material and process parameter in ambient conditions. Effective properties such as modulus of elasticity (E) and thermal conductivity found from characterization should be loaded in FE simulation software to check variation in simulation results compared to actual lattice geometry simulation. Hence, FE software benchmarking is also performed in this work.

### 2.3. Distortion Measurement and Benchmarking FE Simulation for Distortion Prediction

**Figure 4:**
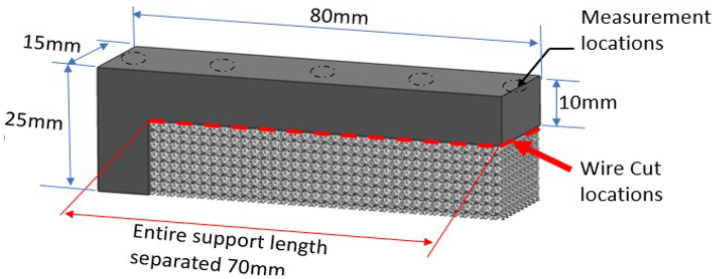
Cantilever test sample with perforated block support for benchmark study.

A benchmarking study is conducted to understand the ability of software to compute distortion and compare it with experimental results to validate the distortion prediction accuracy of commercial simulation software (Simufact Additive). A cantilevered overhang sample with perforated block support geometry, as shown in Figure 4, was selected for the study. The support structure was designed with a VF of 0.3 using Materialize Magics software. After printing, the part–support interface was separated along the cantilever length using wire-cut EDM. The parts distortion after support separation is measured along cantilever length using a Zeiss prismo coordinate measuring machine (CMM), Bangalore, India; three sets of readings were measured at regular intervals of 20 mm, along the length of the cantilever sample. The part–base plate interface was not separated for easy distortion measurements in CMM. The part–base plate joint will help anchor the part at one end and distort at opposite ends upon support removal.

**Figure 5:**
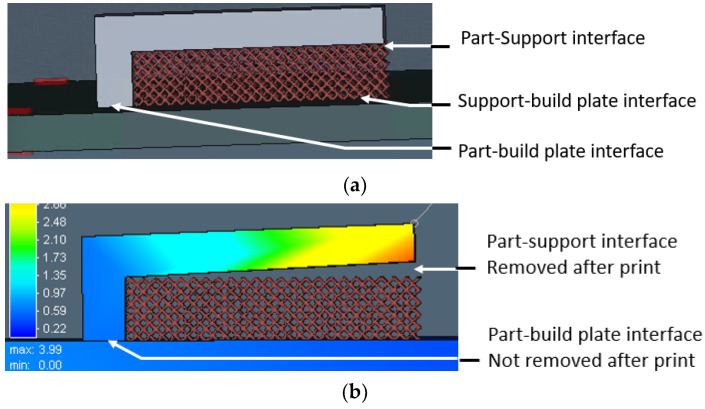
FEA details to simulate experimental steps of support removal. (**a**) Boundary condition in FE model. (**b**) FE simulation result after support removal.

Distortion of the test sample was also analyzed in AM-FEA software using the same support geometry and process parameters from the actual printed sample. The default simulation step in AM-FEA software is base plate removal after printing and followed by support separation from the printed part, which is achieved by deleting all contact points generated between interfaces shown in Figure 5a. For the current study, the simulation steps and boundary conditions were modified by deleting the base plate removal step. Only support interface is removed from part and base plate interface to mimic actual distortion condition measured in CMM as shown in Figure 5b. There is a limitation in AM-FEA software’s boundary condition modification; the entire support is separated from part interface in one step, whereas in, actual hardware, the wire EDM cut is done gradually from the end; this might introduce some variation in final distortion values. 

**Figure 6:**
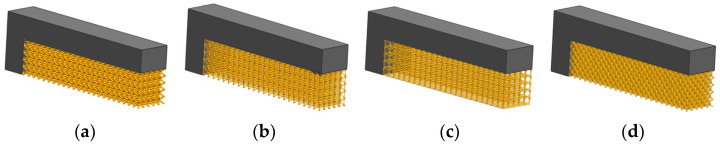
CAD model of cantilever test samples with various lattice supports. (**a**) BCC. (**b**) BCCZ. (**c**) Octahedroid. (**d**) Dodecahedron.

Since the current work focuses on reducing the computational effort involved in distortion prediction, the initial behavior of support structure homogenization is studied by replacing full support geometry with the homogenized model. Equivalent material property for the homogenized model was loaded with modified strength and thermal conductivity with 0.3 VF i.e., $E_{\mathrm{Eff}}$ = 0.3 E and $K_{\mathrm{Eff}}\;$ = 0.3 K. After validation of FE simulation capability, multiple lattice topologies are analyzed using FE simulations and support characterization to understand the influence of various lattice design parameters on part distortion [22]. Thus, five different lattice support topologies were selected for the study. In this work, several standard lattice support geometries are individually characterized, and its effective bulk material property for homogenized equivalent solid support is determined experimentally. Cantilever test samples with different lattice support geometry were designed using the computer-aided design (CAD) software package Unigraphics NX as shown in Figure 6 and printed using the same process parameters.

**Figure 7:**
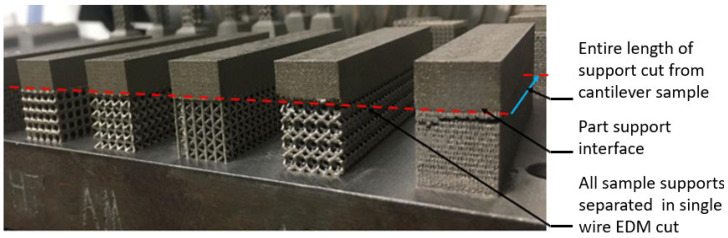
Printed cantilever sample showing the location for support separation.

Printed sample support structures are separated from parts using the same steps from the benchmark study. Since all samples are printed in the single baseplate, all parts need to be aligned perfectly, and wire EDM needs to pass through all part support interfaces in a single pass, as shown in Figure 7. Only the support region is separated from the part and is still anchored to the base plate for distortion measurement. The same steps were analyzed in FE simulation with actual support geometry and homogenized solid support. Homogenized geometry is loaded with effective strength from experimental characterization of modulus of elasticity and effective thermal conductivity calculated using Equation (1). Finally, results are compared to estimate variation in distortion behavior.

After validating software accuracy and distortion from various support topologies, the effect of different lattice geometry design parameters, mainly variation in lattice unit cell size and VF of support, are analyzed using FE simulation by varying actual CAD geometries. Based on the response curve graph generated, an attempt is made to develop a correlation between the design parameter and its effect on cantilever sample distortion. It is used to build a common scaling factor for a given lattice topology that can be used to scale effective properties found from experimental characterization of modulus of elasticity and thermal conductivity. The perforated block cantilever test sample was also printed with a changed VF of 0.15 to validate the findings.

### 2.4. Lattice Support Characterization

**Figure 8:**
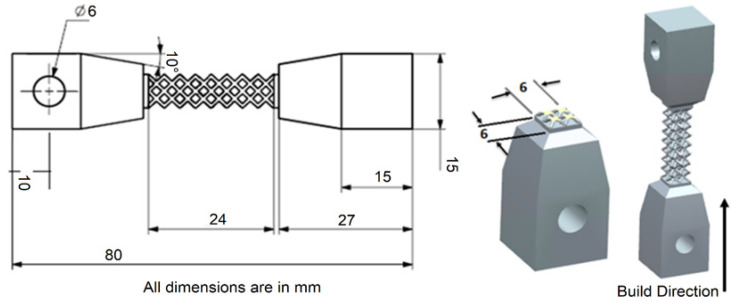
CAD representation and dimensions of tensile test specimen design.

Tensile test samples with square cross-sections are designed using modifications from standard ASTM E8/E8M specimen to suit lattice cell arrangement [23]. A similar square cross-section sample is used by Koehnen et al. [24] and Maskery et al. [25] for lattice geometry characterization. The cross-section of the specimen is modified to 6 mm × 6 mm to have an even distribution of lattice cells. In addition, 24 mm of gauge height is selected. Furthermore, 3 mm lattice cells are designed for all support topology to have an even distribution of whole lattice cells, as shown in Figure 8. Specimens were provided with a 6 mm hole, 90° offset for auto-centering of the sample. This will prevent any torsional stress on the lattice structure.

**Figure 9:**
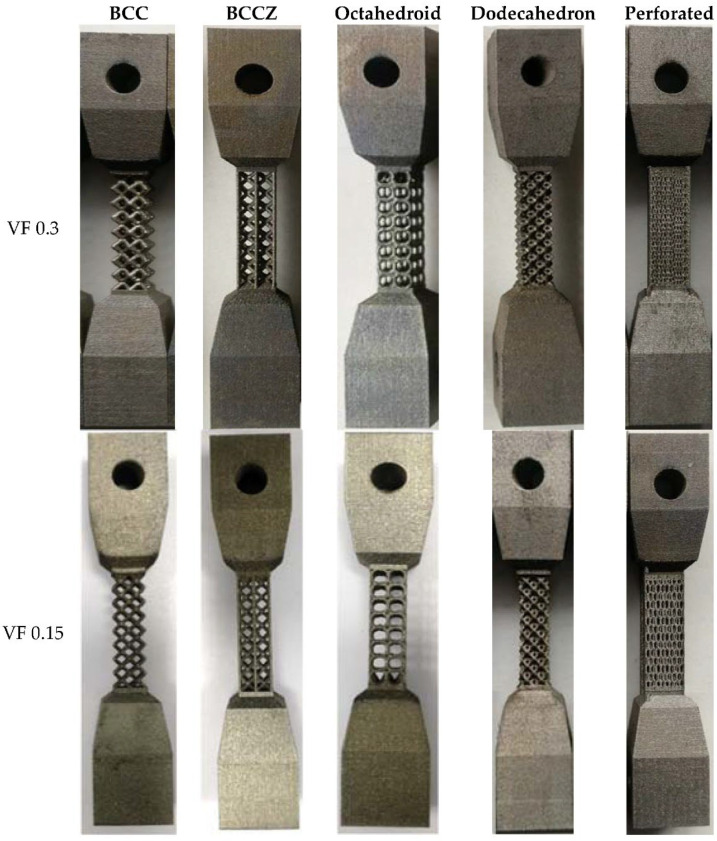
Printed test sample with two different volume fractions.

Five types of lattice tensile samples with two different volume fractions 30 and 15% (shown in Figure 9) are tested to get tensile data. Uniaxial tensile tests were conducted at room temperature using a BISS-Nano plug-and-play Servo hydraulic universal test machine with 15 kN load cell capacity and 1 mm/min strain rate used as per ASTM E8/E8M standard [26]. The strength of complete solid material is taken from the EOS material datasheet generated from the same standard process parameters and EOS recommended material. The experimental results of deformation and load were used to calculate the effective strength property of lattice using Equation (2), where $E_{\mathrm{eff}}\;$ is the effective strength modulus of lattice support, $\sigma_{\mathrm{eff}}\;$ is the effective stress developed, A is the area of lattice cross-section, ΔL is deformation, and L is initial length:

(2) $$E_{\mathrm{eff}}=\frac{\sigma_{\mathrm{eff}}}{Ԑ}=\frac{F/A}{\Delta L/L}$$

The effective strength modulus of lattice samples found from the stress–strain curve is divided by Young’s modulus of 100% solid support to arrive at the effective strength modulus ratio. The calculated ratio is loaded as strength knockdown factor in the homogenized model FEA. Except for BCCZ, all selected lattices have identical geometry in all three directions. BCCZ exhibits high anisotropic behavior in the Z direction due to the presence of extra vertical rods. Based on tested material data from EOS [17], there is approximately 15% stiffness reduction in the Z direction, causing anisotrophy in all selected lattices. For the current study, influence of anisotrophy is neglected, and lattice is assumed to be isotropic by choosing the worst-case value of Z direction, introducing error into the homogenized model. Post validation of the methodology, the effective property is scaled to unknown VF using a suitable scaling function to validate the scalability of the proposed method.

## 3. Results

**Figure 10:**
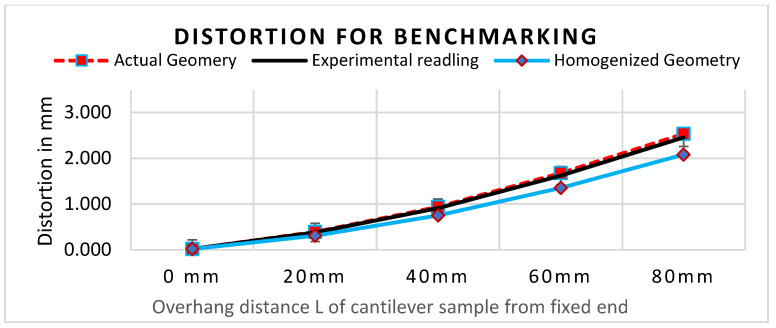
Comparison of distortion measurement without characterization data using only VF.

The benchmark study of FE software accuracy for distortion prediction using perforated block and the equivalent solid block is shown in Figure 10. The comparison of the simulation result (red dotted line in Figure 10) with experimental measurement (black line in Figure 10) shows good agreement with less than 10% error. This result validates the distortion prediction capability of software for actual support geometry considered in the study.

Distortion comparison between VF knockdown homogenized model and experimental reading shows that error is greater than 15%. Thus, direct use of VF as an effective property knockdown factor in the homogenized model does not provide an accurate result. This initial study reveals that the support structure’s effective property depends on other lattice design parameters. Therefore, the support characterization data are used in the homogenized model simulation.

**Figure 11:**
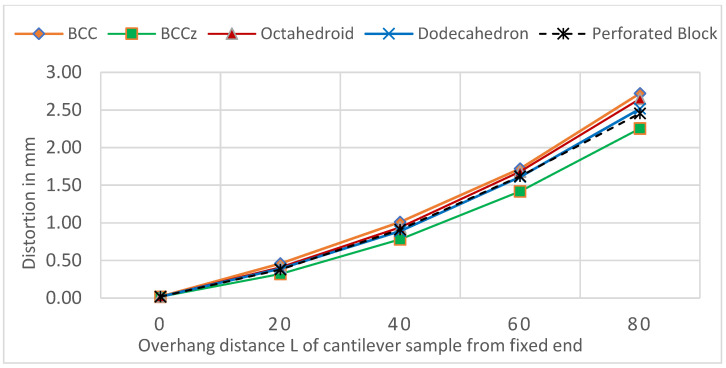
Cantilever part distortion comparison after support separation.

Figure 11 shows the cantilever part distortion variation due to various lattice support topologies. The cantilever part geometry and process parameter were maintained same for all samples to eliminate any possible influence of part geometry on residual stress-led distortion. Wire EDM for support separation was carried on all models together in a single pass to ensure that the support removal process does not contribute to distortion variations. Experimental distortion comparison show that support structure topology will influence the distortion on final part. BCC and BCCZ distortion results comparison shows that adding strut element aligned in a vertical z print direction will increase support structure strength and reduce distortion. BCC and dodecahedron distortion comparison show that adding more crosslinked strut elements will reduce the part distortion. The traditional perforated block support provides good support to minimize part distortion compared to the dodecahedron, BCC, and octahedron, and this may be due to smaller cell size compared to the rest of the lattice geometry; however, closer visual inspection from Figure 7 shows that perforated block support will produce cracks on edges and part–support interface. This may result in print failure for larger components where residual stresses are high. From the selected five support topologies, BCCZ support has the least part distortion and BCC has the worst part distortion. Thus, a comparison of all four lattice support samples shows that geometry with more crosslinked strut elements and strut in the vertical z printing direction will provide better performance against part distortion.

**Figure 12:**
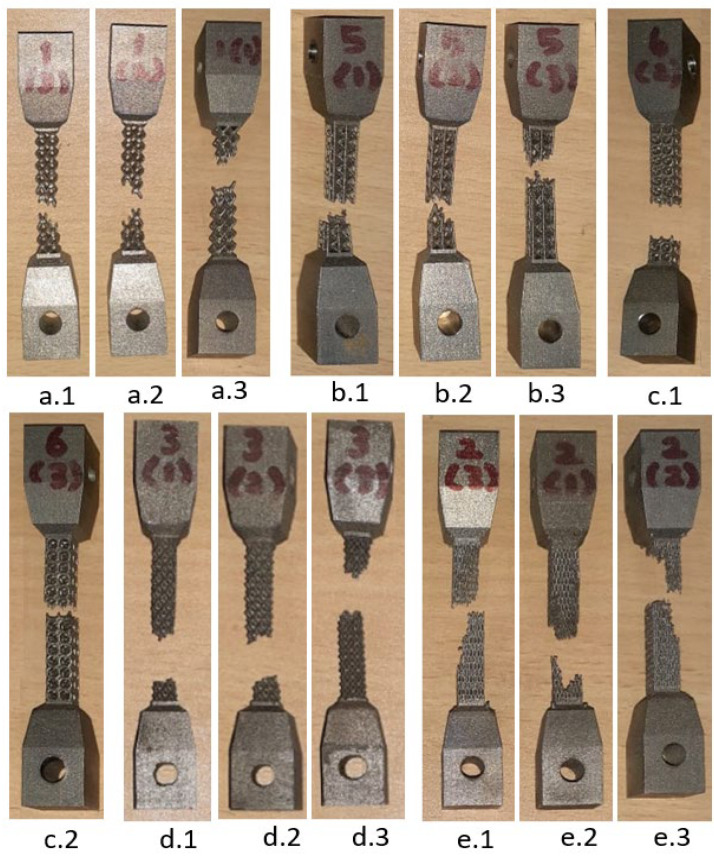
UTM test samples’ failure locations for various lattice geometry.

Tensile test samples from lattice support characterization are shown in Figure 12. It is observed that test coupons made from the same material and same volume fraction exhibit different failure patterns due to their different lattice topology. Shear plane failure with 45° is visible in BCC, BCCZ, and Dodecahedron samples. The perforated block failure region experienced an uneven section with traces of crack growth in the failure plane. The octahedron sample shows failure in the horizontal plane without any noticeable cross-section variation. In addition, the perforated block and octahedron did not show necking in the failure region.

**Figure 13:**
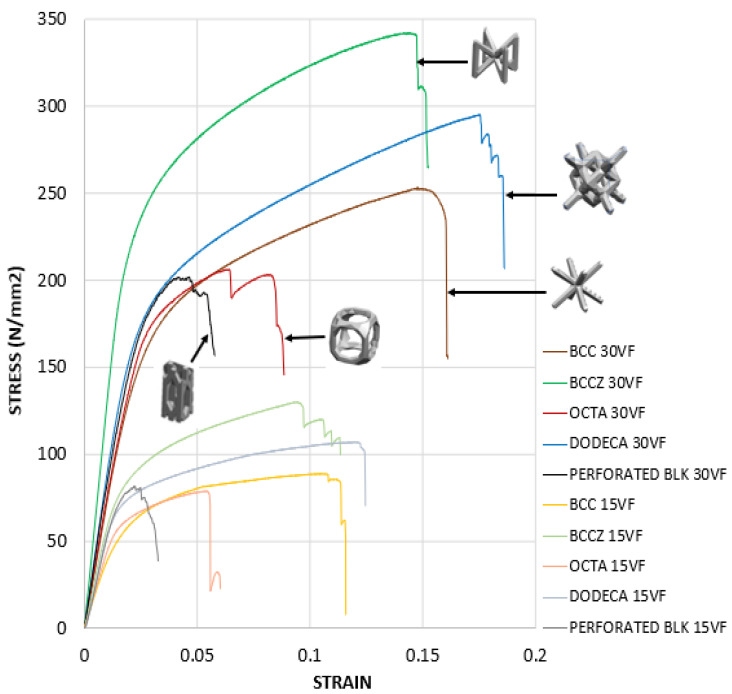
Stress–strain curve of Lattice support test specimen, printed with 0.3 volume fraction.

**Figure 14:**
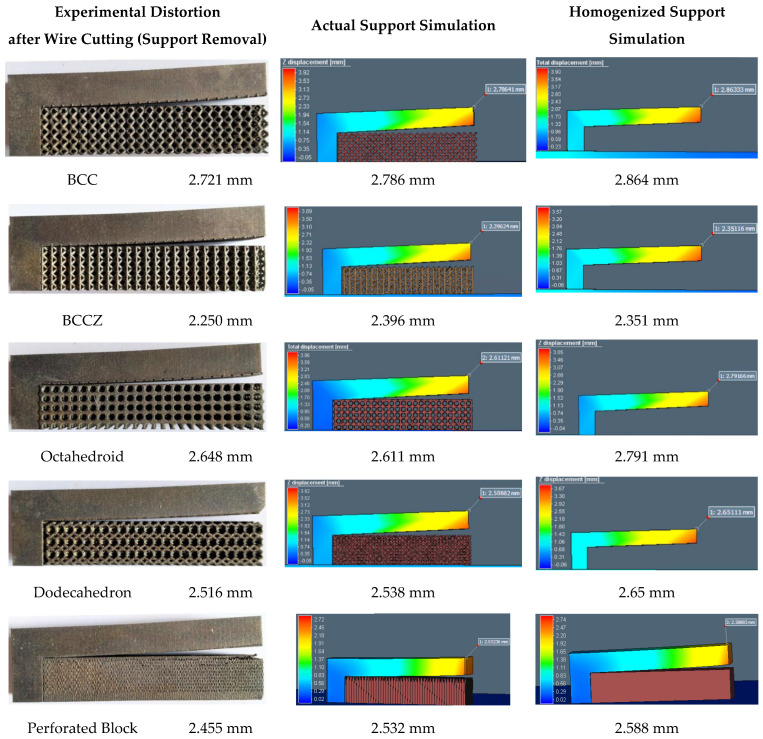
Simulation plots of homogenized and actual support geometry.

**Figure 15:**
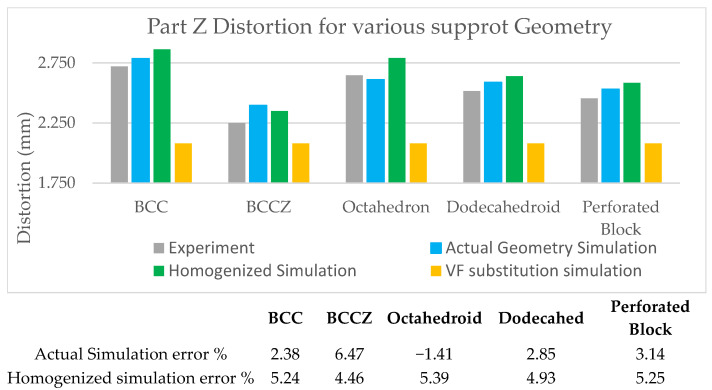
Cantilever part distortion comparison—Experimental versus Simulation results.

The stress–strain curve of lattice support specimen in Figure 13 shows that all selected lattice support structures exhibit a continuous transition from elastic to plastic region without distinguishing yield point. Thus, stress at lattice support deformation/strain by 0.2% is considered as the elastic limit for yield strength. The linear elastic region is used for calculating effective strength modulus, using Equation (2) and presented in Table 3. The effective strength modulus of each lattice support sample found from the stress–strain curve is divided by the strength modulus of 100% solid support to find the effective strength ratio. This ratio is loaded as a strength knockdown factor in the homogenized model simulation. The FE simulation results using effective strength are compared against actual support simulation and experiments in Figure 14 and Figure 15.

FE simulation results comparison shows that the average deviation of homogenized support simulation is approximately 5% compared to actual support simulation of roughly 3%. The use of true support geometry simulation is not consistent. Its under-predicted distortion in the octahedron support and high distortion in BCCZ. On the other hand, the homogenization model is consistent in capturing the behavior of all different lattice topologies. The experimental result and FE simulation error percentage are approximately constant for all topologies except BCCZ support. Thus, loading effective property from characterization will eliminate any variation caused by process variation and provide a consistent result for any selected lattice topology. BCCZ is not isometric in lattice topology. Assuming it as isometric in homogenization has led to the error in the proposed method.

**Figure 16:**
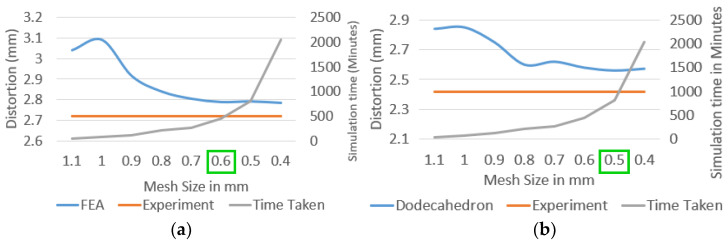

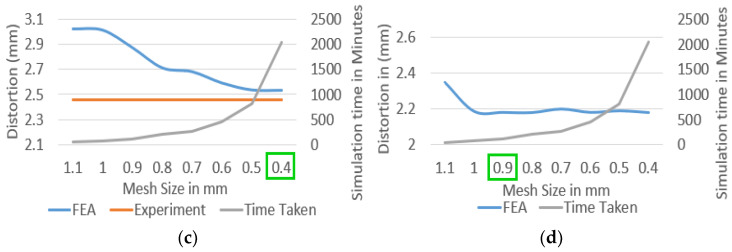
AM simulation mesh sensitivity analysis on the cantilever test model with: (**a**) BCC support; (**b**) dodecahedron support; (**c**) perforated block support; (**d**) homogenized CAD support.

The FE simulation speed comparison between actual geometry and homogenized model can be observed from the mesh sensitivity analysis study presented in Figure 16. Homogenized solid block with single continuum geometry saturates below 0.9 mm mesh. In contrast, it is below 0.5 mm mesh size for actual geometry simulation. i.e., roughly 30% smaller than the minimum size feature. Thus, simulation time comparison shows that the homogenized support models are approximately 6–7 times faster than the actual lattice support geometry.

**Figure 17:**
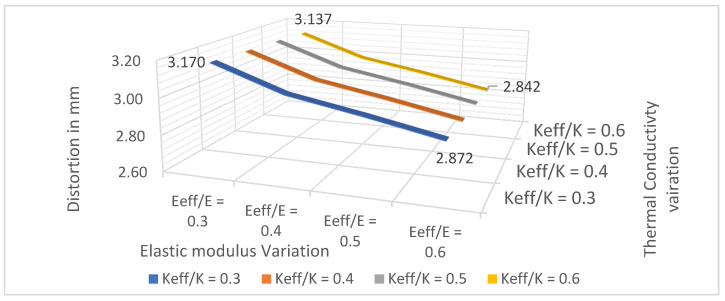
Effective property sensitivity study on a homogenized model to understand FEA software.

The influence of effective modulus and effective thermal conductivity variation on FEA residual stress can be understood from a sensitivity study done by varying E_eff_/E ratio and K_eff_/K ratio on the cantilever sample with homogenized support. AM-FEA package uses a property knockdown factor on both strength modulus and thermal conductivity. Figure 17 shows the plot of cantilever part distortion value for variation in effective modulus and effective thermal conductivity. When effective modulus is varied from E_eff_/E 0.30 to 0.40, its influence on part distortion is approximately 7.5 times the effect of varying K_eff_/K from 0.30 to 0.40. Thus, FEA software is more sensitive to effective modulus variation compared to effective thermal conductivity variation. Therefore, the current study focuses on experimental characterization for effective moduli for various lattice topologies and uses generic effective thermal conductivity for all lattice topologies.

## 4. Discussion

### 4.1. Distortion for Various VF Using Actual Lattice Geometry in AM Simulation

**Figure 18:**
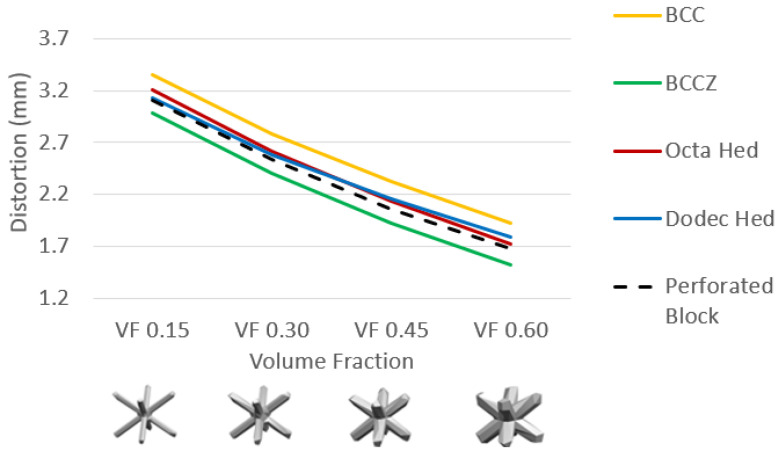
Actual geometry FE simulation for varying support volume fraction.

The distortion of various cantilever beams for VF variation is found using the actual geometry of AM simulation and presented in Figure 18. FE results show that support geometries show a similar slope for volume fraction change. Variation in the slope height for different support topologies shows that separate experimental characterization needs to be performed when new lattice topologies are selected. Subsequently, it can be scaled to various VF using a suitable scaling factor fixed for lattice topology. Therefore, the error due to VF change on the homogenized model is negligible, which provides an opportunity to scale specific experimental findings to any VF without the need to repeat experiments.

### 4.2. Scaling of Effective Property

To scale the effective property of a lattice support to various volume fractions, the Gibson–Ashby model [27] is used to develop mathematical relations between the volume fraction of lattice support and its effective property using the following equations:

(3) $$\frac{\tilde{E}}{E_{s}}=C1{\left[\frac{\tilde{\rho }}{\rho_{s}}\right]}^{m}$$

where $\tilde{E}\;$ is strength modulus of porous lattice, $E_{s}$ strength modulus of complete solid material, $\tilde{\rho }$ is density of porous lattice, and $\rho_{s}$ is density of complete solid material. According to the Gibson–Ashby model, the relative modulus of strength is proportional to relative VF using proportionality coefficients m and C1 [27]. Equation (3) is a power function, so m and C1 are found by converting the power function to a linear function by taking the logarithm on both sides and using at least two VF conditions. Based on the study by Sharma et al. [28], power function relation “m” is decided based on whether the lattice strut experiences bending-dominated behavior or stretching-dominated behavior, so Maxwell stability criteria is verified on all selected lattice topologies.

Table 4 shows that all lattice geometry experiences bending-dominated behavior with details of calculation on Maxwell number can be referred in work done by Deshpande et al. [29]. Thus, for a bending-dominated open-celled metal lattice, the relative modulus of strength is proportional to the square (m = 2) of relative density [30]. However, when Equation (3) is substituted with a fixed value of m = 2 for characterized conditions of VF 0.30 and 0.15, the C1 value does not match both cases. Thus, m and C1 are found by converting Equation (3) power function to a linear function by taking the logarithm on both sides and using two known VF conditions.$\rho_{s}$

**Figure 19:**
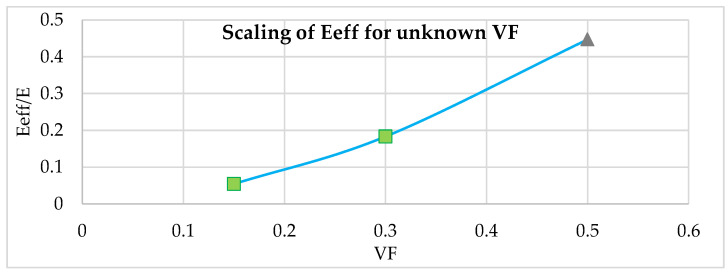
Effective property of scaled effective property using the Gibson–Ashby model.

Perforated block support is modeled at VF = 0.15, 0.30 and 0.5 using the scaled effective property from Table 5, $\frac{{\;E}_{\mathrm{Eff}}}{E}$ of 0.054, 0.183 and 0.447, respectively. The grey triangle marker in Figure 19 and gray cells in Table 5 is scaled using experimental results from green square marker in Figure 19 or green cells in Table 5. Results comparisons are shown in the following Table 6. These three conditions are selected to verify the scalability of effective property to unknown VF. Here, green-colored cells are experimental data found from characterization, and grey-colored cells are scaled effective properties using Equation (3). These selected points are parallelly simulated using true geometry simulation and effective property homogenized simulation.

The results comparison shows that the error between homogenized model FE results and experimental distortion measurement is 6.2% and 5.4% for VF of 0.15 and 0.30, respectively. When the effective property from VF 0.15 and 0.30 is scaled to VF 0.5, the error between homogenized model FE results and experimental distortion measurement is found to be in an acceptable range of 10.4%.

## 5. Conclusions

This work modeled cantilever test samples with various lattice support topologies and printed successfully for part distortion measurement. Experimental characterization of tensile test samples was performed to find the effective modulus of different lattice support topologies. The effective property findings from experiments and analytical calculations are used in a simple homogenized solid model to predict part distortion. Subsequently, the effective property was scaled to unknown VF and validated for one lattice topology. The main conclusions from the present study are:

- Results show that substituting complex lattice geometry with a homogenized solid has increased simulation speed by 6–7 times.
- The error between homogenized model simulation and experiment results is consistent, whereas actual geometry simulation accuracy depends on the shape selected; this is the advantage of experimental characterization and can capture geometry-dependent printing variations.
- FE and experiment result comparisons of scaled VF show good agreement with approximately less than 10% variation. This provides evidence for the scalability of the proposed methodology.
- Effective thermal conductivity used in this study by a generic equation fails to capture the effect of various lattice topologies, providing scope for future improvement to find actual thermal conductivity from the experimental method.

## Figures and Tables

**Table 1:** Chemical composition of Inconel718 powder (IN718).

Nickel (Ni)	Chromium (Cr)	Iron (Fe)	Cobalt (Co)	Aluminum (Al)	Molybdenum (Mo)	Niobium (Nb)	Titanium (Ti)	Silicon (Si)
53.20%	18.91%	17.96%	0.15%	0.37%	3.04%	5.14%	0.92%	0.08%

**Table 2:** IN718 process parameter details for EOS M280.

Process Parameter	Units	Value
Laser Power	W	285
Scanning speed	mm/s	960
Hatch Spacing	mm	0.11
Size of Laser Beam	mm	0.3
Lag between layer	seconds	10
Layer thickness	μm	40
Scan strategy	–	10 mm Strips 67° rotation on each layer

**Table 3:** Mechanical properties of support structures.

Support Structure	Sample VF	0.2% Yield Stress (N/mm^2^)	Ultimate Tensile Stress (N/mm^2^)	Effective Modulus E_eff_ (N/mm^2^)	Ratio E_eff_/E
BCC	0.30	172.2	251.6	24,480	0.153
	0.15	68.2	91.2	7246	0.045
BCCZ	0.30	201.2	341.5	38,880	0.243
	0.15	86.4	130.7	14,240	0.089
Octahedroid	0.30	172.5	205.3	26,240	0.164
	0.15	72.3	79.8	6960	0.043
Dodecahedron	0.30	173.5	293.8	28,960	0.181
	0.15	74.6	123.2	9140	0.057
Perforated Bock	0.30	179.5	198.5	29,280	0.183
	0.15	78.5	80.4	8780	0.054

**Table 4:** Maxwell’s stability criteria check.

	Struts (s)	Nodes (N)	Maxwell Number M	Remarks
BCC	8	9	−13	Bending-dominated
BCCZ	12	9	−9	Bending-dominated
Octahedroid	12	8	−6	Bending-dominated
Dodecahedron	32	21	−25	Bending-dominated

**Table 5:** Scaling relation using the Gibson–Ashby model for perforated block support.

Perforated Block (VF)	Gibson–Ashby Coefficients		E_eff_	Knockdown Factor E_eff_/E
	C1	m		
0.15	1.51	1.75	8730	0.054
0.30	1.51	1.75	29287	0.183
0.45	1.51	1.75	59549	0.372
0.60	1.51	1.75	98542	0.616
0.50	1.51	1.75	71612	0.447

**Table 6:** VF of homogenized model scaled using the Gibson–Ashby model for perforated block support.

Experimental Distortion		Actual Support Simulation	Homogenized Support FEA
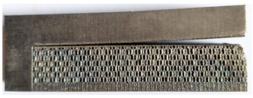		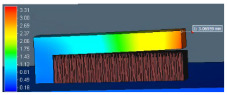	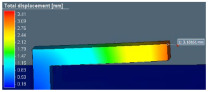
VF 0.15 perforated	2.993 mm	3.070 mm	3.181 mm
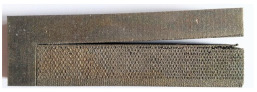		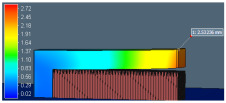	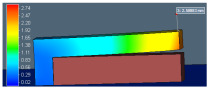
VF 0.30 perforated	2.455 mm	2.532 mm	2.588 mm
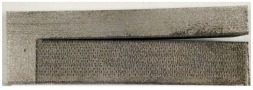		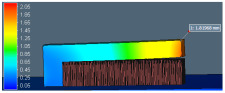	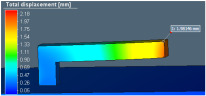
VF 0.50 perforated	1.794 mm	1.819 mm	1.981 mm (With scaled Eeff)
